# Multiscale intracranial EEG dynamics across sleep–wake states: toward memory-related processing

**DOI:** 10.3389/fncom.2025.1618191

**Published:** 2025-10-24

**Authors:** Juan M. Tenti, Monserrat Pallares Di Nunzio, Marisa A. Bab, Osvaldo Anibal Rosso, Fernando Montani, Marcelo J. F. Arlego

**Affiliations:** ^1^Instituto de Investigaciones Fisicoquímicas Teóricas y Aplicadas (INIFTA), Universidad Nacional de La Plata (UNLP)-Consejo Nacional de Investigaciones Científicas y Técnicas (CONICET), La Plata, Argentina; ^2^Facultad de Ciencias Exactas, Universidad Nacional de La Plata, La Plata, Argentina; ^3^Instituto de Física La Plata (IFLP) - Consejo Nacional de Investigaciones Científicas y Técnicas (CONICET), Departamento de Física, Universidad Nacional de La Plata, La Plata, Argentina; ^4^Instituto de Física (UFAL), Maceio, Brazil; ^5^Facultad de Ciencias Exactas, Universidad Nacional del Centro de la Provincia de Buenos Aires, Tandil, Argentina

**Keywords:** sleep, memory consolidation, intracranial EEG, neural dynamics, multiscale analysis, cortical regions, temporal correlations, avalanche dynamics

## Abstract

Sleep is known to support memory consolidation through a complex interplay of neural dynamics across multiple timescales. Using intracranial EEG (iEEG) recordings from patients undergoing clinical monitoring, we characterize spectral activity, neuronal avalanche dynamics, and temporal correlations across sleep-wake states, with a focus on their spatial distribution and potential functional relevance. We observe increased low-frequency power, larger avalanches, and enhanced long-range temporal correlations—quantified via Detrended Fluctuation Analysis—during N2 and N3 sleep. In contrast, REM sleep and wakefulness show reduced temporal persistence and fewer large-scale cascades, suggesting a shift toward more fragmented and flexible dynamics. These signatures vary across cortical regions, with distinctive patterns emerging in medial temporal and frontal areas—regions implicated in memory processing. Rather than providing direct evidence of consolidation, our results point to a functional neural landscape that may favor both stabilization and reconfiguration of internal representations during sleep. Overall, our findings highlight the utility of iEEG in revealing the multiscale spatio-temporal structure of sleep-related brain dynamics, offering insights into the physiological conditions that support memory-related processing.

## 1 Introduction

Understanding the neural dynamics that characterize different states of consciousness, such as wakefulness, non-rapid eye movement (NREM) sleep, and rapid eye movement (REM) sleep, is crucial for elucidating how the brain establishes conditions conducive to memory consolidation. Previous work has established hallmark electrophysiological signatures across vigilance states—posterior dominant alpha during restful wakefulness, slow waves and sleep spindles in NREM (especially N2/N3), and REM-specific patterns such as sawtooth waves and regionally localized delta activity (Berry et al., 2012; Troester et al., 2023; Steriade et al., 1993; De Gennaro and Ferrara, 2003; Fernandez and Lüthi, 2020; Frauscher et al., 2020). Yet most studies isolate a single domain or scale; a unified framework that jointly quantifies spectral content, scale-dependent temporal structure, and spatial organization within the same intracranial dataset remains under development.

This work investigates how neural signals across wakefulness and sleep exhibit state-specific dynamics that may create a functional environment favorable for memory-related processes, employing a multiscale and multimodal approach. Using intracranial EEG (iEEG) recordings from the Multicenter iEEG Sleep Atlas (von Ellenrieder et al., 2020), we apply complementary methods—including spectral analysis, neuronal avalanche statistics, and detrended fluctuation analysis (DFA)—to examine how brain activity patterns, as operationally defined by sleep stages, vary across time scales and anatomical regions.

Studies have demonstrated that sleep stages exhibit distinct neural features. For instance, slow oscillations in the delta band during NREM sleep have been associated with synaptic downscaling, potentially facilitating the stabilization of neural circuits involved in declarative memory (Diekelmann and Born, 2010; Rasch and Born, 2013). Sleep spindles, characteristic of NREM sleep, have been implicated in the facilitation of synaptic plasticity, thereby supporting the consolidation of motor skills and procedural memories (Fogel and Smith, 2011). Conversely, REM sleep has been associated with the reactivation and reorganization of memory traces—processes that may contribute to the integration of emotional content and the refinement of neural representations (Walker, 2009).

Beyond traditional spectral methods, neuronal avalanche analysis enables the characterization of spontaneous cascades of activity across time and space. Empirical observations show that avalanche size and duration distributions display approximate scale-invariant behavior across wake, NREM, and REM sleep (Ribeiro et al., 2010; Wilting and Priesemann, 2019). These patterns have been interpreted in various theoretical frameworks, but here we focus on characterizing how avalanche properties vary empirically across states.

In addition, Detrended Fluctuation Analysis (DFA) is introduced here as one of three principal analytical techniques to quantify long-range temporal structure in iEEG signals, providing complementary insights into the scale-dependent dynamics of neural activity.

By integrating frequency-specific spectral signatures, neuronal avalanche statistics, and DFA-derived temporal structure, this study offers a multiscale characterization of brain dynamics across sleep–wake states, with potential implications for sleep-dependent memory processing and neural organization.

## 2 Materials and methods

### 2.1 The atlas dataset

In this study, we utilized data from the Multicenter iEEG Sleep Atlas (ATLAS). This international collaboration has compiled a comprehensive open-access dataset of intracranial EEG (iEEG) recordings from patients with drug-resistant focal epilepsy. Importantly, these recordings encompass both wakefulness and various sleep stages, curated to reflect physiologically normal activity (von Ellenrieder et al., 2020).

The iEEG Sleep ATLAS provides an opportunity to examine cortical dynamics during NREM (N2, N3), REM, and waking states across 38 distinct brain regions, including frontal, temporal, parietal, occipital, and insular cortices. Through precise anatomical localization and expert sleep scoring, this dataset enables the exploration of how different cortical areas contribute to state-dependent neural activity patterns associated with sleep processes.

The recordings consist of artifact-free, 60-second epochs selected during resting wakefulness (eyes closed) and each annotated sleep stage. Signals were resampled at 200 Hz and filtered (0.5–80 Hz). Note that these preprocessing steps are part of the ATLAS pipeline; infra-slow activity (< 0.5 Hz) is therefore unavailable. Analyses are reported in 0.7–75 Hz to ensure robust estimates within the 0.5–80 Hz acquisition bandwidth (fs = 200 Hz). The standardized ATLAS format facilitates reproducibility and integration with computational pipelines, supporting analyses of local and distributed activity patterns. Representative raw iEEG examples across wake, N2, N3, and REM are available from the ATLAS reference used here (e.g., Figure 1A in Kalamangalam et al., 2021).

The richness and granularity of this dataset are particularly suited for investigating neural dynamics at multiple spatiotemporal scales. Specifically, it allows for comparisons of regional activity by means of measures such as power spectra, neuronal avalanches, and temporal correlations across sleep–wake states, particularly those implicated in memory consolidation.

Table 1 summarizes the number of channels per brain region and lobe across sleep and waking conditions. While electrode coverage varies across subjects—particularly during sleep—this variability is addressed by conducting the analysis at the population level. This approach allows us to identify region-specific trends and explore how different cortical areas may contribute to the neural dynamics associated with memory processes.

**Table 1:** The 38 regions classified by brain lobes, and the number of channels per region for the non-REM N2, N3, and REM sleep states, and the waking state.

**No. Reg**	**Brain region**	**Lobe**	**No. channels W-N2-N3-R**
1	Superior and middle occipital gyri	Occipital	21 - 16 - 16 - 14
2	Inferior occipital gyrus and occipital pole	Occipital	23 - 22 - 22 - 17
3	Cuneus	Occipital	19 - 18 - 18 - 18
4	Calcarine cortex	Occipital	12 - 10 - 10 - 8
5	Lingual gyrus and occipital fusiform gyrus	Occipital	29 - 21 - 21 - 12
6	Postcentral gyrus (including medial segment)	Parietal	64 - 43 - 43 - 31
7	Superior parietal lobule	Parietal	53 - 40 - 40 - 36
8	Parietal operculum	Parietal	41 - 31 - 31 - 10
9	Supramarginal gyrus	Parietal	70 - 65 - 65 - 39
10	Angular gyrus	Parietal	53 - 52 - 52 - 42
11	Precuneus	Parietal	43 - 37 - 37 - 32
12	Posterior cingulate	Parietal	29 - 25 - 25 - 14
13	Anterior insula	Insular	71 - 54 - 54 - 35
14	Posterior insula	Insular	35 - 25 - 25 - 14
15	Gyrus rectus and orbital gyri	Medial frontal	45 - 41 - 41 - 28
16	Anterior cingulate	Medial frontal	31 - 31 - 31 - 19
17	Middle cingulate	Medial frontal	40 - 31 - 31 - 21
18	Supplementary motor cortex	Medial frontal	47 - 37 - 37 - 28
19	Medial frontal cortex	Medial frontal	19 - 15 - 15 - 10
20	Central operculum	Medial frontal	63 - 46 - 46 - 29
21	Frontal operculum	Lateral frontal	29 - 24 - 24 - 18
22	Opercular part of inferior frontal gyrus	Lateral frontal	38 - 30 - 30 - 20
23	Triangular part of inferior frontal gyrus	Lateral frontal	47 - 41 - 41 - 29
24	Orbital part of inferior frontal gyrus	Lateral frontal	19 - 17 - 17 - 13
25	Middle frontal gyrus	Lateral frontal	173 - 149 - 149 - 106
26	Superior frontal gyrus and frontal pole	Lateral frontal	89 - 78 - 78 - 64
27	Medial segment of superior frontal gyrus	Medial frontal	16 - 16 - 16 - 13
28	Medial segment of precentral gyrus	Medial frontal	18 - 13 - 13 - 11
29	Precentral gyrus	Lateral frontal	123 - 82 - 82 - 60
30	Superior temporal gyrus	Temporal	79 - 69 - 69 - 46
31	Middle temporal gyrus	Temporal	126 - 115 - 115 - 73
32	Inferior temporal gyrus	Temporal	41 - 40 - 40 - 29
33	Temporal pole and planum polare	Temporal	22 - 16 - 16 - 7
34	Transverse temporal gyrus	Temporal	14 - 12 - 12 - 4
35	Planum temporale	Temporal	43 - 29 - 29 - 15
36	Fusiform and parahippocampal gyri	Temporal	45 - 41 - 41 - 29
37	Hippocampus	Temporal	36 - 30 - 30 - 13
38	Amygdala	Temporal	6 - 6 - 6 - 5

Counts are pooled across the entire dataset, considering all available 60-s epochs in each state.

### 2.2 Spectral analysis

In this section, we provide a brief overview of the spectral analysis technique applied in this work. The primary tool utilized in this section is the *power spectral density* (PSD), which is defined as the Fourier transform of the autocorrelation function (Semmlow and Griffel, 2014). For discrete signals, such as those found in the ATLAS, denoted as *x*[*n*],


(1) $$\begin{array}{l} \text{PSD}\left[m\right]=\overset{N}{\underset{n=1}{\sum }}r_{xx}\left[n\right]\exp\left(-\frac{i2\pi mn}{N}\right),\;m=0,1,\ldots ,N/2, \end{array}$$

in which *N* is the total number of samples and *r*_*xx*_[*n*] is the autocorrelation function for a discrete-time signal. This function indicates the similarity of a signal with a delayed copy of itself as a function of the delay time *k*. It is defined by


(2) $$\begin{array}{l} r_{xx}\left[k\right]=\frac{1}{N}\overset{N}{\underset{n=1}{\sum }}x\left[n\right]x\left[n-k\right]. \end{array}$$

Alternatively, the spectral power PSD[*m*] can be calculated using the Parseval relation


(3) $$\begin{array}{l} \text{PSD}\left[m\right]=|X\left(m\right){|}^{2}, \end{array}$$

where *X*[*m*] is the discrete Fourier transform (Semmlow and Griffel, 2014):


(4) $$\begin{array}{l} X\left[m\right]=\frac{1}{N}\overset{N-1}{\underset{n=0}{\sum }}x\left[n\right]\exp\left(-\frac{i2\pi mn}{N}\right),\;m=0,1,\ldots ,N-1, \end{array}$$

in which $\frac{2\pi }{N}$ is the spacing between samples.

In general, when dealing with biosignals, we typically have only a sample of a longer signal; therefore, spectral analysis becomes an estimation process. A commonly employed approach is to compute the Power Spectral Density (PSD) over multiple segments of the sample. These segments can then be averaged to generate a spectrum with improved overall characteristics. When the PSD is computed directly using the Fourier Transform (FT) followed by averaging, it is referred to as a *periodogram*. One of the most widely used methods for calculating the average periodogram is the Welch technique (as seen, for example, in Semmlow and Griffel, 2014). In this approach, overlapping segments are employed, and a shaping window (i.e., a non-rectangular window) is applied to each segment. Traditional periodograms typically average the spectra of overlapping segments, usually with a 50% overlap. Averaging introduces a trade-off between spectral resolution, which decreases with averaging, and statistical reliability. We report results both with and without frequency-wise normalization. In the normalized case, the PSD at each frequency are rescaled by the corresponding across-epoch mean, which emphasizes relative shifts and crossover points across conditions. The unnormalized PSD, in contrast, preserve the absolute power distribution.

To estimate band-specific features, we followed the procedure of Kalamangalam et al. (2020). For each 60 s epoch, the power spectrum was smoothed with a Savitzky–Golay filter to reduce high-frequency noise while preserving local spectral structure (Virtanen et al., 2020). The smoothed spectrum was then modeled as the sum of five Gaussians (delta, theta, alpha, beta, gamma), fitting amplitude, center frequency, and bandwidth (σ) for each component. Fits were accepted only if *R*^2^ ≥ 0.95. All spectral estimates were restricted to 0.7–75 Hz; bands were defined as delta (0.7–4 Hz), theta (4–8 Hz), alpha (8–12 Hz), beta (12–30 Hz), and gamma (30–75 Hz). This procedure yields robust, reproducible estimates while minimizing contamination by noise or non-oscillatory features.

### 2.3 Neuronal avalanches

In terms of iEEG activity, a neuronal avalanche is defined as a consecutive series of time slots with a width of Δ*t* that contains at least one active electrode (Beggs and Plenz, 2003; Plenz and Thiagarajan, 2007). Each avalanche is preceded and followed by at least one time slot without activity. The size of an avalanche is determined by the sum of the absolute amplitudes of the iEEG signal at the active electrodes or simply by the number of active electrodes (*s*). An electrode is considered active when the signal exceeds a certain threshold. In this study, we identify avalanches in the iEEG signals of the Atlas using the number of active electrodes, separated by periods of inactivity, as a measure of avalanche size.

Specifically, for each electrode, the signal was normalized by subtracting its mean and dividing by its standard deviation, resulting in a z-scored signal with zero mean and unit variance. Discrete events were defined as individual time points where the normalized signal crossed below a threshold of −2.0 standard deviations. This thresholding strategy is widely used to detect statistically significant deflections from baseline activity, while minimizing the influence of background fluctuations and noise (Plenz and Thiagarajan, 2007; Beggs and Plenz, 2003).

Binary event trains were constructed from these threshold crossings, and the activity was subsequently binned into consecutive, non-overlapping 10 ms time windows—a commonly used window size in the literature for capturing scale-invariant neuronal dynamics (Beggs and Plenz, 2003). A bin was considered active if at least one electrode registered an event within that window. A neuronal avalanche was then defined as a sequence of contiguous active bins, bounded before and after by at least one empty bin. The avalanche size was computed as the total number of events (across all electrodes) occurring within the sequence.

The distribution of avalanche sizes is a key indicator of scale-invariant behavior in complex systems. Empirical distribution of avalanche sizes allows the characterization of scaling behavior in brain activity. Previous empirical work shows that avalanche sizes often follow approximate power-law distributions across species and human data (Ribeiro et al., 2010; Priesemann et al., 2013). In this work we focus on the operational definition and empirical estimation of avalanche statistics.

We analyzed the statistical properties of avalanche sizes and durations by fitting their probability density functions (PDFs) to power-law models. Specifically, the PDF of avalanche sizes, *P*(*s*), was fitted to:


(5) $$\begin{array}{l} P\left(s\right)\propto s^{-\tau }, \end{array}$$

where τ is the exponent governing the decay pattern of the size distribution. Similarly, the PDF of avalanche durations, *P*(*T*), was fitted to:


(6) $$\begin{array}{l} P\left(T\right)\propto T^{-\alpha }, \end{array}$$

where α is the exponent shaping the duration distribution.

In addition, we examined the scaling relationship between the average avalanche size, 〈*s*〉(*T*), and its corresponding duration *T*:


(7) $$\begin{array}{l} \langle s\rangle \left(T\right)\propto T^{\gamma }, \end{array}$$

where γ is the scaling exponent that relates size and duration (Friedman et al., 2012). The value of γ was obtained via linear regression on logarithmically transformed data.

To estimate this exponent, we fitted the avalanche size distributions using the discrete maximum likelihood method implemented in the powerlaw Python package (Alstott et al., 2014), which provides robust estimation for power-law exponents in empirical datasets. The value of *x*_min_, defining the lower cutoff above which power-law behavior is assumed, was selected as the one that provides the best compromise between the distributions of avalanche exponents across all sleep states, aiming to minimize systematic biases in the comparison between them.

A fixed temporal binning was used to discretize the neural activity time series, independent of the overall activity level. This approach ensures consistent time resolution across different sleep stages, avoiding potential biases introduced by adaptive binning methods that may conflate changes in activity rate with changes in the temporal structure of avalanches. In addition, we quantified the mean activity per active bin, defined as the total number of detected events divided by the number of bins in which at least one channel registered an event, thereby capturing the average event rate conditional on periods of activity.

Note that we use τ for the avalanche *size* exponent and α for the avalanche *duration* exponent. The symbol α is also used in Sections 2.4–3.3, 4.3 for the DFA scaling exponent; meanings are disambiguated by context (avalanche duration vs. DFA) and by explicit references in captions and text.

Finally, we use the compact format *x* = *a*(*b*) to denote *x* = *a*±*b* in the last digit(s). For example, α = 0.84(2) means 0.84 ± 0.02. Unless noted otherwise, *b* is one standard error of the estimate. The same notation is employed for DFA scaling exponent estimations.

### 2.4 Detrended fluctuation analysis

In this study, we use Detrended Fluctuation Analysis (DFA) to assess temporal correlations in iEEG signals from the ATLAS, particularly focusing on scaling properties.

The steps for calculating the DFA in this work are as follows (Hardstone et al., 2012):

- Calculate the cumulative sum (signal profile): The cumulative sum {*X*_*i*_} is computed by successively summing the values of the iEEG signal {*x*_*j*_} and subtracting the mean value 〈*x*〉:
(8) $$\begin{array}{l} X_{i}=\overset{i}{\underset{j=1}{\sum }}\left(x_{j}-\langle x\rangle \right). \end{array}$$
  In Equation 8, *x*_*j*_ denotes the *j*-th sample of the demeaned discrete iEEG signal within a 60-s epoch; *X*_*i*_ is the cumulative-sum (profile) up to index *i*. Here *i* ∈ {1, …, *N*} is the running index of the profile, *j* is the summation index, and *N* is the number of samples in the epoch.
- Define windows: A set of window sizes, denoted as Δ*t*, is defined. These sizes are evenly distributed on a logarithmic scale, ranging from a minimum of four samples to the length of the signal.
- Divide the cumulative sum into *N* overlapping windows: A window of length Δ*t* is chosen, and a series of consecutive windows {*w*_*n*_(Δ*t*)} of the same size is created from the signal profile, with a 50% overlap.
- Remove linear trend in each window: For each window *w*_*n*_(Δ*t*), fit a straight line (trend) using a least-squares fit and subtract it from the window to create the detrended window *w*_*nD*_(Δ*t*):
(9) $$\begin{array}{l} w_{nD}\left(\Delta t\right)=w_{n}\left(\Delta t\right)-{\text{Linear-Trend}}_{n}. \end{array}$$
- Calculate the standard deviation of detrended windows: For each detrended window *w*_*nD*_(Δ*t*), calculate the standard deviation (Fluctuation Function *F*(Δ*t*)):
(10) $$\begin{array}{l} F\left(\Delta t\right)=\sqrt{\frac{1}{N}\overset{N}{\underset{n=1}{\sum }}{\left[w_{nD}\left(\Delta t\right)-\langle w_{nD}\left(\Delta t\right)\rangle \right]}^{2}}. \end{array}$$
- Repeat for different window sizes: Iterate the above steps for all window sizes Δ*t*.
- Plot log(*F*(Δ*t*)) vs. log(Δ*t*): The slope of this log-log plot (estimated via linear regression) yields the DFA exponent α.

The scaling exponent α characterizes the scaling properties of mean fluctuations across time scales in the iEEG signal. As described in Hardstone et al. (2012):

α < 0.5: Anti-correlated time series (smaller fluctuations at larger scales).
α = 0.5: Uncorrelated (random) process.
0.5 < α < 1: Positively correlated time series.
1 < α < 2: Non-stationary process.

In this work, we use the term long-range temporal correlations (LRTC) strictly for the stationary regime 0.5 < α < 1 (Hardstone et al., 2012). Values α ≈ 1 correspond to 1/*f*-like (pink) scaling, whereas α > 1 indicate non-stationary, integrated dynamics; in the latter case, long-range dependence typically characterizes the increments of the process rather than the raw signal itself (Poil et al., 2012; Dalla Porta and Copelli, 2019). This convention is adopted to avoid conflating stationary LRTC with non-stationary persistence.

## 3 Results

### 3.1 Spectral analysis of wake and sleep stages

To assess how different stages of the sleep-wake cycle modulate brain dynamics we performed spectral analysis on iEEG data using power spectral density (PSD) estimates computed via Welch's method (600-sample windows, 50% overlap). Both absolute and normalized PSDs were analyzed globally and by cortical region. Details of the preprocessing and spectral estimation pipelines are provided in Section 2.2.

#### 3.1.1 Global spectral profiles

**Figure 1:**
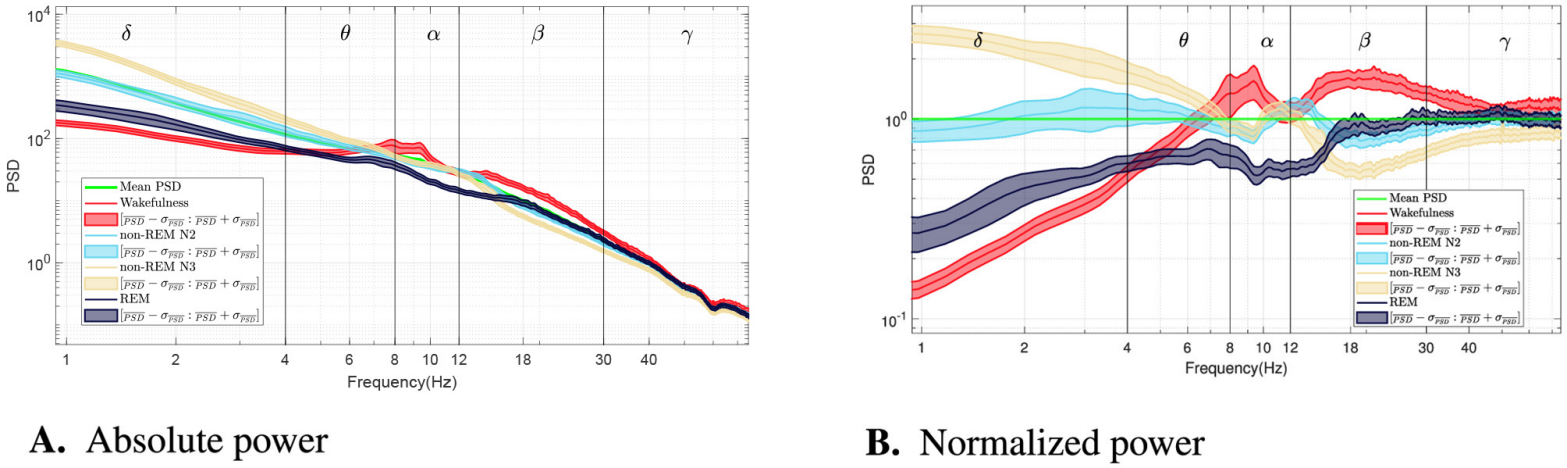
**(A)** Grand-average power spectral density (PSD) for each vigilance state (log–log scale), obtained by averaging across all 60-s epochs in the dataset; the green line shows the across-epoch mean at each frequency (pooled across states). **(B)** Same spectra as the left panel, after normalizing each curve by the across-epoch mean at each frequency, highlighting relative shifts and crossover points. During sleep, there is a significant increase in delta band power around 1 Hz, indicating non-REM sleep. Theta band activity increases during wakefulness, particularly in its higher range. Additionally, REM sleep shows a reduction in spectral density between 8 and 20 Hz. Furthermore, non-REM N2 and N3 stages exhibit a distinct peak around 12 Hz in the spectral profile, followed by a drop in the beta band, distinguishing them from wakefulness and REM sleep.

Figure 1 summarizes the global spectral patterns across vigilance states. Figure 1A displays the grand-average PSDs (log–log scale), obtained by averaging across all 60-s epochs in the dataset. Figure 1B shows the same spectra after normalizing each curve by the across-epoch mean at each frequency, emphasizing relative shifts and crossover points.

As expected, non-REM sleep stages—particularly N3—exhibit a marked increase in delta power around 1 Hz, with differences exceeding an order of magnitude compared to wakefulness. Theta activity is most pronounced in wakefulness near 7–8 Hz. A distinct peak around 12 Hz, corresponding to sleep spindles, emerges during N2 and N3. REM sleep shows a broadband reduction in power between alpha and low beta bands (8–20 Hz). In the gamma range, all sleep stages display lower power than wakefulness, though with smaller effect sizes.

#### 3.1.2 Regional spectral features

To assess spectral differences across brain states at the local level, we conducted independent (unpaired) Mann–Whitney (*U*) tests (Virtanen et al., 2020) on Gaussian-fitted amplitudes for canonical frequency bands (delta, theta, alpha, beta, and gamma) across predefined lobes (see Table 1). We compared (i) Wake vs REM and (ii) N2 vs. N3. To control for multiple comparisons over band–lobe combinations, we applied the Benjamini–Hochberg false discovery rate (FDR) procedure (Benjamini and Hochberg, 1995) at α = 0.05.

The contrast between Wake and REM revealed focal differences mainly in alpha amplitude in temporal, parietal and occipital cortices, and delta differences in frontal regions. Conversely, the N2 vs. N3 comparison exhibited more widespread and robust differences, especially in beta and gamma bands, with temporal, frontal, and parietal regions showing strong modulation. All reported differences correspond to statistically significant contrasts, with FDR-corrected *p*-values below 0.05.

To further explore spatial heterogeneity in spectral organization, we examined region-specific PSDs. Several representative cases are highlighted below.

**Figure 2:**
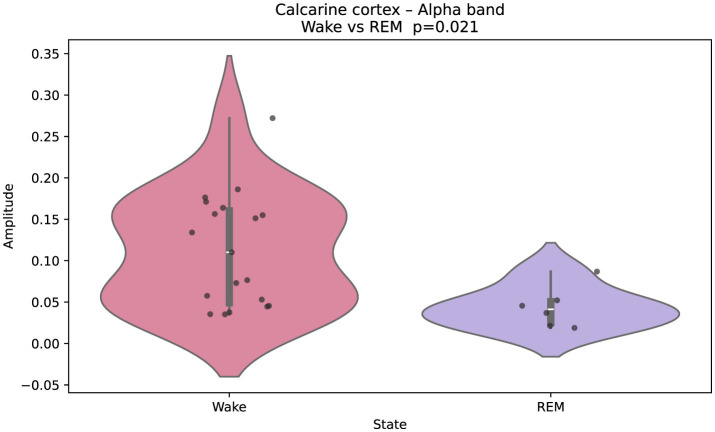
Gaussian-fitted alpha-band amplitudes in the calcarine cortex (primary visual cortex) during wakefulness and REM sleep. Higher alpha power in wakefulness reflects the characteristic occipital alpha rhythm, attenuated in REM. Black points indicate individual single-epoch (60 s) Gaussian fits; violin plots summarize their distribution for each state. Group differences were assessed with a two-sided Mann–Whitney U test; multiple comparisons were controlled via FDR. The same plotting convention is used in Figures 3–5.

**Figure 3:**
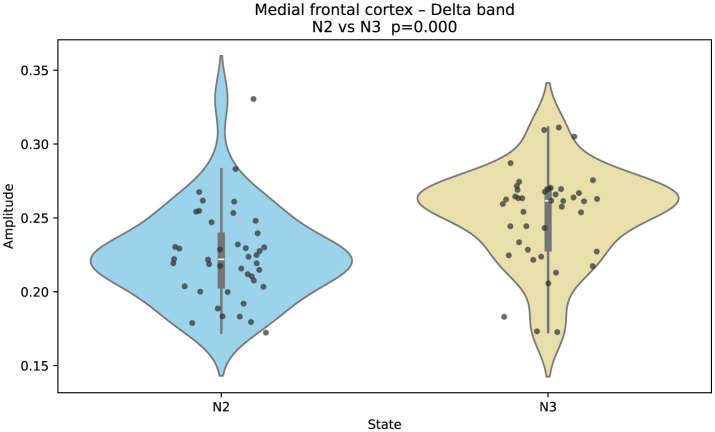
Gaussian-fitted delta-band amplitudes in the medial frontal cortex across sleep stages N2 and N3. Delta amplitude increases significantly in N3 relative to N2 (two-sided Mann–Whitney U; FDR-controlled). This pattern reflects enhanced cortical slow-wave synchrony during deep sleep in frontal-medial regions.

**Figure 4:**
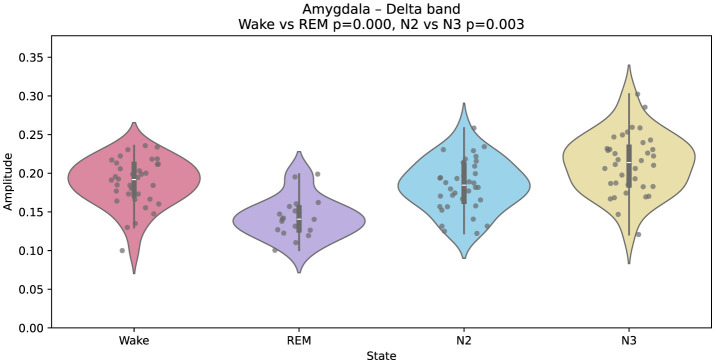
Gaussian-fitted delta-band amplitudes in the amygdala across wakefulness, REM, N2, and N3. A relative decrease in delta power is observed during REM compared to wakefulness, suggesting distinct modulatory dynamics in this limbic structure.

**Figure 5:**
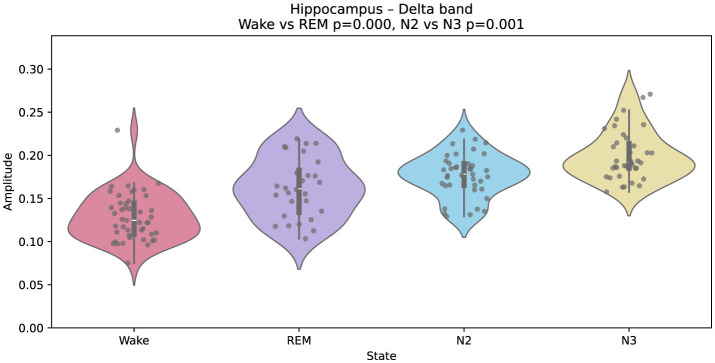
Gaussian-fitted delta-band amplitudes in the hippocampus across wakefulness, REM, N2, and N3. Unlike most cortical regions, delta amplitude is higher during REM than wakefulness.

Figure 2 illustrates Gaussian-fitted alpha-band amplitudes in the calcarine cortex (primary visual area). Black points denote single-epoch (60 s) Gaussian fits, and violins summarize their distribution across epochs for each state. A clear separation is observed, with higher alpha during wakefulness and reduced amplitude during REM. The same plotting convention is used in Figures 3–5.

On the other hand, Figure 3 shows that Gaussian-fitted delta-band amplitudes in the medial frontal cortex increase in N3 relative to N2. This elevation reflects enhanced slow-wave synchrony in frontal-medial regions during deep sleep.

Interestingly, the amygdala exhibits a different spectral pattern. As shown in Figure 4, delta amplitude is lower during REM than during wakefulness, diverging from the cortical trend. This result aligns with previous reports of limbic-specific modulation during REM, potentially related to emotional processing and memory reorganization (Kalamangalam et al., 2021).

A similar divergence is found in the hippocampus (Figure 5), where delta amplitude is higher during REM than during wakefulness. This contrasts with most cortical and subcortical areas and supports the view that the hippocampus exhibits unique REM-related dynamics. Prior studies have linked such patterns to memory consolidation mechanisms and the replay of waking experiences (Kalamangalam et al., 2021; von Ellenrieder et al., 2020).

We present a lobe-level overview with selected, illustrative region-level PSDs, emphasizing the most challenging contrasts (Wake vs. REM; N2 vs. N3). An exhaustive region-by-region survey is beyond the scope of this work.

### 3.2 Neuronal avalanches across wakefulness and sleep states

Here, we investigate neuronal avalanches—spontaneous cascades of neural activity—across wakefulness and sleep stages using intracranial EEG data from the iEEG Atlas. Our analysis focuses on quantifying how avalanche size distributions vary systematically with vigilance state, without assuming a specific underlying dynamical regime.

#### 3.2.1 Neuronal avalanche results

Neuronal avalanches were identified using a discrete event-based approach, following the established methodology of binning thresholded neural activity in short time windows (Section 2.3).

Given the variability in the number of implanted electrodes across patients, we observed that the power-law scaling of avalanche size distributions was often disrupted in subjects with lower electrode coverage. To mitigate the inevitable bias introduced by spatial sub-sampling, only patients with at least 16 electrodes were included in the analysis. For each selected patient, a uniform subset of 16 electrodes was considered; in cases where more than 16 electrodes were available, a random selection was performed to ensure comparability across subjects. This approach reduces variability due to differences in electrode coverage and allows for a more consistent estimation of avalanche statistics across patients.

Avalanche size distributions were fitted using the discrete maximum likelihood method implemented in the powerlaw package, which estimates the power-law exponent τ and the exponential cutoff parameter λ. To ensure consistency across all conditions and avoid statistical instabilities associated with very small avalanches, the lower cutoff *x*_min_ was fixed at 3. This choice excludes the smallest events, which are often dominated by noise or are undersampled due to the temporal discretization, and focuses the analysis on more reliable segments of the avalanche size distribution. Similar fixed thresholds have been employed in previous studies to enhance the robustness of power-law fitting and cross-condition comparisons.

**Figure 6:**
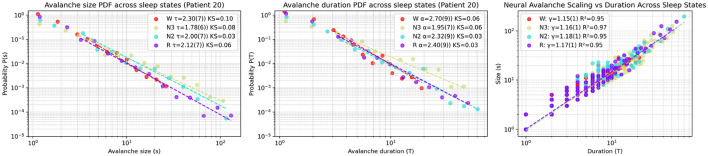
Representative avalanche statistics across sleep stages for Patient 20. **(Left)** Probability density function (PDF) of avalanche sizes (s), fitted to power-law distributions with exponents τ and Kolmogorov-Smirnov (KS) distances quantifying the goodness-of-fit for each sleep stage: Wake (W), REM (R), N2, and N3. **(Middle)** PDF of avalanche durations (T), similarly fitted to power laws with exponents α and corresponding KS distances. **(Right)** Scaling relation between mean avalanche size (〈*s*〉) and duration (T) in log-log scale, with linear regression fits yielding slopes γ (indicating the scaling exponent) and *R*^2^ values assessing the fit quality.

The analysis used Patient 20 as an example of the statistical procedure, showing distinct avalanche dynamics across sleep stages in Figure 6. The probability density function (PDF) of avalanche sizes (*s*) followed power-law distributions with exponents τ as follows: Wake [τ = 2.30(7), KS = 0.1], REM [τ = 2.12(7), KS = 0.06], N2 [τ = 2.00(7), KS = 0.03], and N3 [τ = 1.78(6), KS = 0.08], with Kolmogorov-Smirnov (KS) distances indicating fit quality. The PDF of avalanche durations (*T*) also showed power-law behavior with exponents α: Wake [α = 2.70(9), KS = 0.06], REM [α = 2.40(9), KS = 0.03], N2 [α = 2.32(9), KS = 0.03], and N3 [α = 1.95(7), KS = 0.06], supported by KS distances. The scaling relation between mean avalanche size (〈*s*〉) and duration (*T*) in log-log scale was linear, with slopes γ: Wake [γ = 1.15(1), *R*^2^ = 0.95], REM [γ = 1.17(1), *R*^2^ = 0.95], N2 [γ = 1.18(1), *R*^2^ = 0.95], and N3 [γ = 1.16(1), *R*^2^ = 0.97], and *R*^2^ values confirming a consistent trend across sleep states. These results suggest varying neural avalanche properties depending on sleep stage.

The scaling exponent τ—estimated as the slope of the fit in log–log space—indicates a higher frequency of large avalanches during non-REM sleep relative to REM and wakefulness.

**Figure 7:**
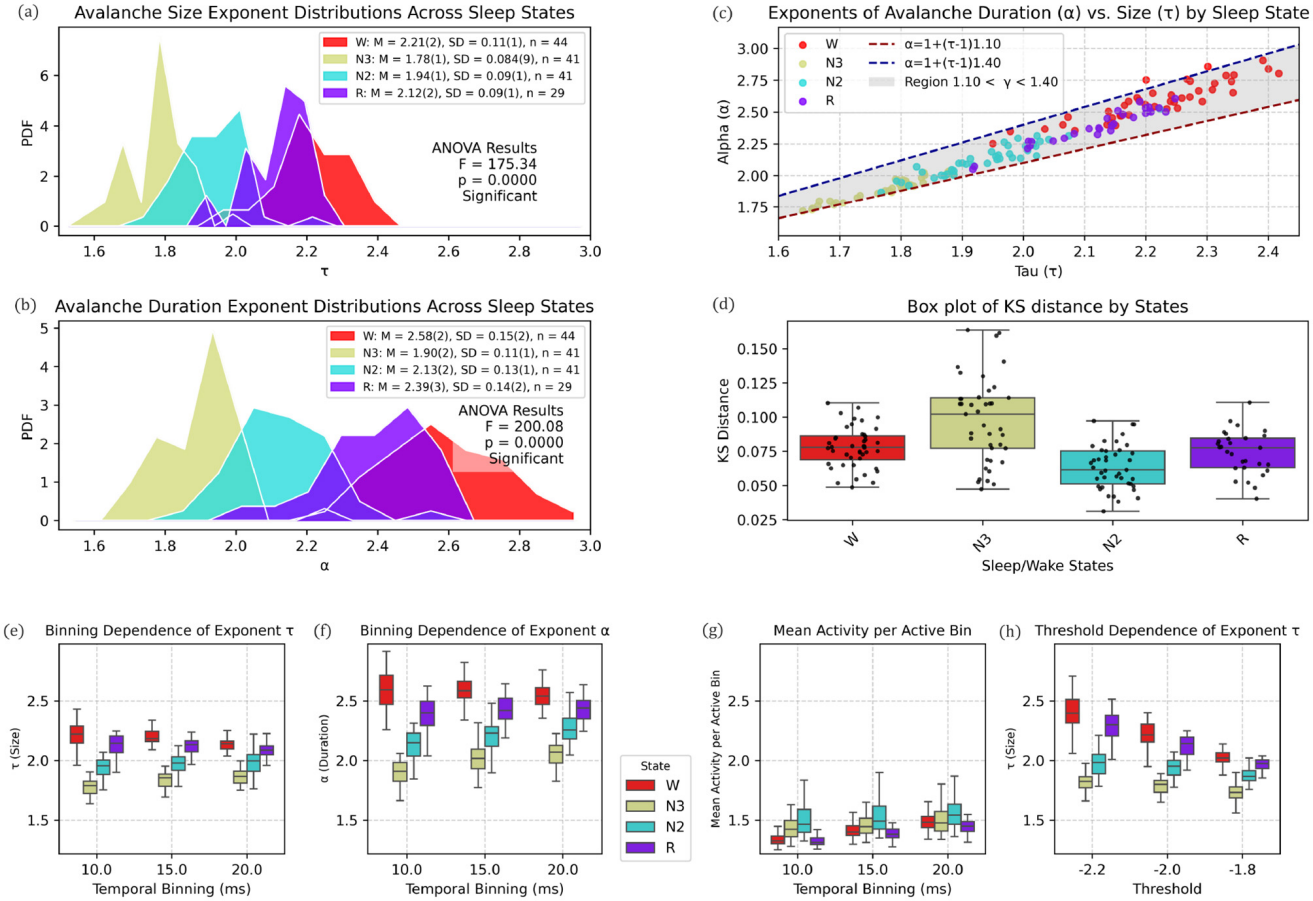
State-dependent modulation of avalanche scaling exponents across sleep stages. **(a)** Probability density functions (PDFs) of avalanche size exponents (τ) for vigilance states Wake (W, red), N3 (yellow), N2 (cyan), and REM (purple). Group means (M), standard deviations (SD), and sample sizes (*n*) are shown in the inset, together with one-way ANOVA results (*F* = 175.34, *p* < 0.0001), indicating significant differences across states. **(b)** PDFs of avalanche duration exponents (α) for the same states as in **(a)**. Both means and ANOVA results (*F* = 200.08, *p* < 0.0001) show a systematic decrease in α with increasing sleep depth. **(c)** Relationship between α and τ for all subjects, color-coded by vigilance state. Theoretical predictions α = 1+(τ−1)γ for γ = 1.10 (red dashed) and γ = 1.40 (blue dashed) are plotted, with the shaded region covering 1.10 < γ < 1.40. The exponent γ increases from ~1.10 in N3 and N2, through intermediate values in REM, to ~1.40 in Wake, reflecting a state-dependent shift in scaling. **(d)** Distributions of Kolmogorov–Smirnov (KS) distances for avalanche size fits across states. Boxplots display medians, interquartile ranges, whiskers, and individual data points. Fits are best (lowest KS) during N2, intermediate in W and R, and poorest in N3, with deeper sleep stages showing stronger deviations from power-law scaling. **(e–h)** Dependence of avalanche measures on analysis parameters. **(e)** The size exponent τ shows modest variation with bin width (10–20 ms), with changes below 5% across all states. **(f)** The duration exponent α is more affected, particularly in N3, where variations approach 9%. **(g)** Mean activity per active bin displays state-dependent differences, being higher in W and R and lower in N2 and N3. **(h)** Changes in detection threshold (−2.2, −2.0, −1.8 SD) shift the absolute values of τ, but the relative ordering of states is preserved, with W and R consistently above N2 and N3.

Extending this analysis to the entire cohort, Figure 7 shows histograms of the scaling size exponent τ (upper left panel) and duration exponent α (middle left panel) across all patients and vigilance states. Avalanche size exponent (τ) values decreased progressively from wakefulness W (*M* = 2.21, *SD* = 0.11, *n* = 44) and REM (*M* = 2.12, *SD* = 0.09, *n* = 29), to N2 (*M* = 1.94, *SD* = 0.09, *n* = 41) and N3 (*M* = 1.78, *SD* = 0.084, *n* = 41). A similar pattern appeared for the duration exponent (α): W (*M* = 2.58, *SD* = 0.15), REM (*M* = 2.39, *SD* = 0.14), N2 (*M* = 2.13, *SD* = 0.13), and N3 (*M* = 1.90, *SD* = 0.11).

Prior to applying one-way ANOVA, assumptions were confirmed: Shapiro–Wilk tests within groups (*n* ≈ 29–44) yielded *p* > 0.05, indicating no departures from normality; Levene's test confirmed homogeneity of variances (*p* ≥ 0.14), conditions under which ANOVA is robust for balanced designs (Zhou et al., 2023; Agbangba et al., 2024; Shaw and Mitchell-Olds, 1993). One-way ANOVA results were highly significant: size exponent (*F* = 175.34, *p* = 0.0000) and duration exponent (*F* = 200.08, *p* = 0.0000), indicating a systematic shift in avalanche dynamics with increasing sleep depth.

To assess the scaling relationship between avalanche size (τ) and duration (α), we calculated the scaling exponent γ from the slope relating τ and α (Figures 7a, b). Across vigilance states, γ values ranged from approximately 1.10 in N3 sleep, increasing through N2 and REM, and reaching about 1.40 during wakefulness. The points followed a clear positive association between τ and α, with all states falling within the reference band (1.10–1.40; Figure 7c).

The KS distance (Figure 7d), quantifying deviations from power-law dynamics in neuronal avalanches, varied significantly across sleep/wake states (Figure 7). N3 exhibited the strongest deviation (median KS = 0.104, IQR: 0.072–0.116), suggesting the poorest fit to power-law scaling. In contrast, N2 showed the closest alignment (median KS = 0.063, IQR: 0.053–0.075), followed by R (0.076) and W (0.079). The broader distribution in N3 (SD = 0.032, max = 0.167) implies state-dependent variability in neuronal dynamics, with deeper sleep stages (N3) departing most markedly from scale-free dynamics.

The bottom row of Figures 7e–h shows the dependence of avalanche measures on temporal binning within the restricted range of 10–20 ms. For the size exponent τ (Figure 7e), mean values changed only slightly across bin widths, with relative variations below 5% in all states (maximum ≈4.8% in N3). The duration exponent α (Figure 7f) showed larger shifts in mean values, with the strongest variation in N3 (≈8.8%) compared to smaller changes in W, R, and N2 (< 6%).

Mean activity per active bin (Figure 7g) exhibited clear state differences in average levels: N2 (1.514-1.585) showed the highest mean activity, followed by N3 (1.457-1.512), whereas W (1.340-1.490) and R (1.326-1.456) were lower (*N*2 > *N*3 > *W* ≈ *R*).

For the threshold dependence of τ (Figure 7h), relative variations were modest in N2 (≈5.6%) and N3 (≈5.9%), but substantially larger in R (≈16.1%) and W (≈19.2%). Despite this increased sensitivity, the relative ordering of states was preserved across thresholds, indicating that the state-dependent hierarchy in τ remains robust to moderate changes in detection criteria.

### 3.3 Detrended fluctuation analysis of sleep and wake states

We applied Detrended Fluctuation Analysis (DFA) to intracranial EEG (iEEG) recordings from the Multicenter iEEG Sleep ATLAS to investigate long-range temporal correlations (LRTCs) across wakefulness and sleep stages. DFA quantifies the presence of LRTCs by estimating a scaling exponent (α), where α = 0.5 indicates uncorrelated noise, 0.5 < α < 1 denotes persistent correlations, and α > 1 suggests non-stationary behavior. Detailed methodological parameters are provided in Section 2.4.

We focused on the intermediate scale range (0.04s–2.5s), which shows the highest variability (as will be shown below) and physiological relevance, in line with previous studies (Hardstone et al., 2012; Meisel et al., 2017; Zhang et al., 2021). Other regions, while potentially interesting, are not the focus here: besides the uniformity of behavior observed both below and above this range, for scales above 2.5s the behavior not only becomes more uniform but also yields poorer statistics due to the limited number of available window sizes inherent to the method. This intermediate range therefore provides sufficient richness to explore meaningful state-dependent differences.

**Figure 8:**
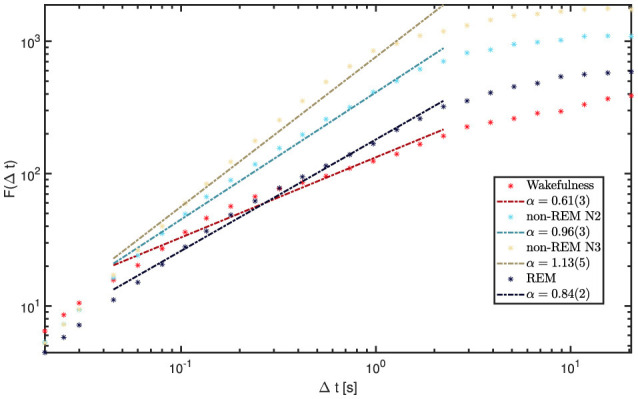
DFA results for wakefulness and sleep stages from channel 1 of a representative patient (9). Short windows (< 0.04 s) and long windows (>2.5 s) show relatively uniform slopes across states. In contrast, the intermediate range (0.04-2.5s) displays the largest variability and the most distinctive state-dependent differences, making it the focus of our analysis. The legend reports the fitted α values for each state within this range.

Each of the 5,720 available iEEG channels (60 s epochs sampled at 200 Hz) was analyzed individually. Figure 8 shows the fluctuation function *F*(Δ*t*) for a representative 60 s epoch. While short time windows (< 0.04s) and very long ones (>2.5s) display relatively uniform patterns across vigilance states, the intermediate range clearly reveals the strongest and most distinctive state-dependent differences, making it the central focus of our analysis.

#### 3.3.1 DFA global features

**Figure 9:**
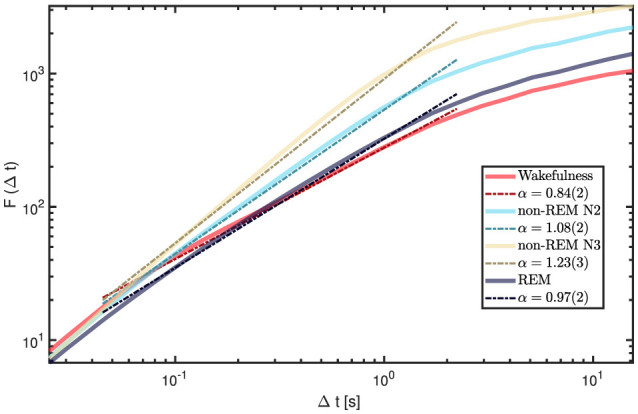
Global DFA curves for each vigilance state (grand-averaged across all channels and all available 60-s epochs per state). The intermediate range (0.04-2.5s) shows the greatest variability across states, with α highest for N3, followed by N2, REM, and wakefulness. The legend reports the fitted α values for each state within this range.

We computed grand-average fluctuation functions by pooling all available 60 s epochs and channels within each state, and then estimated global α exponents by fitting a line in the intermediate window range (0.04s–2.5s). Results are displayed in Figure 9. Wakefulness and REM sleep exhibited α values between 0.8 and 1.0, consistent with stationary LRTC. In contrast, N2 and N3 showed α > 1, indicating non-stationary, integrated dynamics with strong temporal persistence, in line with the predominance of slow oscillations in NREM. To avoid terminological ambiguity, we reserve “LRTC” for the stationary regime 0.5 < α < 1; α > 1 reflects non-stationary persistence (with long-range dependence in the increments), rather than stationary LRTC (Hardstone et al., 2012).

#### 3.3.2 DFA variability across regions

**Figure 10:**
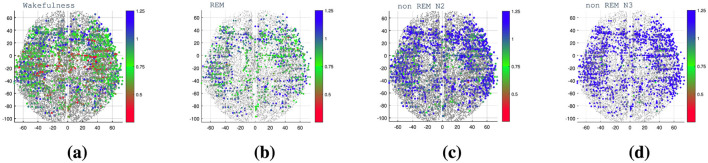
Brain mapping of the DFA exponent (α) depicted in a transverse plane of the brain. The color bar indicates the value of the exponent α. **(a)** Waking state, **(b)** REM, **(c)** non-REM N2, and **(d)** non-REM N3 sleep states. The exponent values across the brain, reveal transitions from wakefulness and REM to non-REM sleep states. Positive correlations characterize wakefulness, while non-REM states show non-stationary behavior. Values near 0.5 represent randomness, with distinct electrode contact locations visible, especially in single-patient data.

To better understand how temporal dynamics distribute across brain regions and sleep stages, we first inspect the spatial patterns of the DFA exponent (α). From Figure 10, it is evident how the DFA exponent reflects the transition from wakefulness and REM to non-REM sleep states, where correlations are positive, moving toward non-stationary states. Only in the wakefulness state can values near 0.5 be observed, indicating a lack of correlations and a tendency toward randomness in the signal. The electrode contact locations (aligned in a straight line) are also discernible, particularly in cases where data are obtained from a single patient.

To quantify these observations, we then calculated, for each lobe and state, key dispersion metrics of the DFA exponent (α), specifically: mean standard deviation (SD), and interquartile range (IQR). SD highlights regions showing greater variability across epochs or subjects, while IQR serves as a robust spread measure less sensitive to outliers. Particularly during deep sleep (N3), regions with elevated SD or IQR may indicate functional heterogeneity or state-dependent modulation.

**Figure 11:**
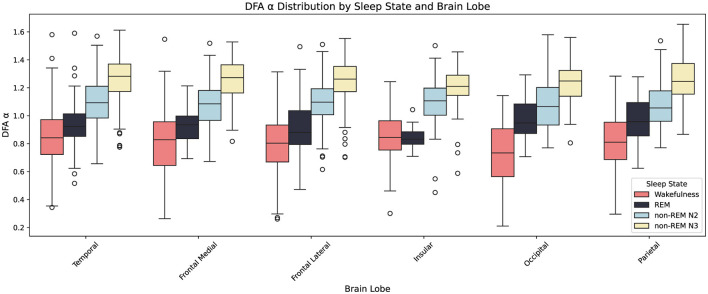
Boxplots showing the distribution of the Detrended Fluctuation Analysis (DFA) exponent α across six brain lobes (Temporal, Frontal Medial, Frontal Lateral, Insular, Occipital, Parietal) and four sleep states (Wakefulness, REM, non-REM N2, non-REM N3). Each box represents the interquartile range (IQR), with the central line showing the median and whiskers extending to 1.5×IQR; outliers are plotted individually. Colors denote sleep states as indicated in the legend. Across most lobes, α increases progressively from wakefulness to deep non-REM sleep (N3). An exception is the insular lobe, which shows no significant difference between Wake and REM.

Figure 11 illustrates the distribution of α across brain lobes and sleep states (Wakefulness, REM, non-REM N2, and non-REM N3). As sleep deepens, α increases monotonically across all lobes, reflecting enhanced temporal persistence and integration.

To evaluate the statistical significance of these inter-state differences, paired Mann–Whitney U tests (Virtanen et al., 2020) were conducted for each lobe comparing Wake vs. REM and N2 vs. N3, with False Discovery Rate (FDR) correction using the Benjamini–Hochberg method (Benjamini and Hochberg, 1995). Table 2 presents the resulting −log_10_(*p*)-values.

**Table 2:** FDR-corrected significance levels (−log_10_*p*) for DFA exponent α comparisons between sleep states by brain lobe.

**Brain lobe**	**Wake vs.REM**	**N2 vs.N3**
Temporal	4.60	32.14
Frontal medial	5.01	21.92
Frontal lateral	7.55	33.45
Insular	(n.s.)	3.74
Occipital	6.10	7.00
Parietal	5.75	24.94

These results indicate that differences between Wake and REM are generally less significant compared to those between N2 and N3. Notably, the insular lobe does not show a significant difference between Wake and REM (denoted “n.s.”), whereas the N2 vs. N3 comparison reveals highly significant differences across most lobes—especially in temporal and frontal lateral regions. This pattern suggests that while deeper sleep stages involve substantial reorganization of cortical temporal dynamics, particularly in medial and temporal areas, the differences between Wake and REM are more subtle and regionally stabilized.

**Figure 12:**
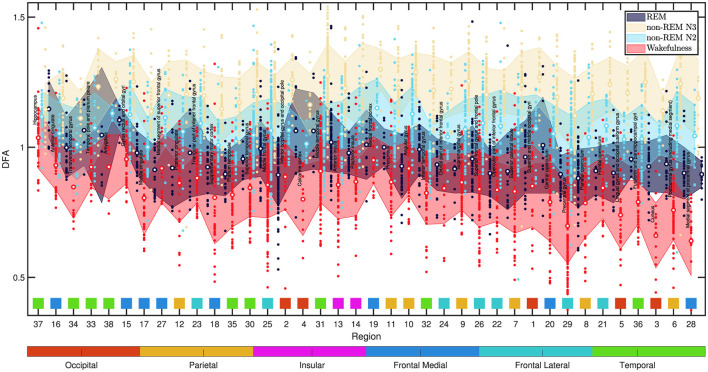
DFA exponent α values across various brain regions are depicted in the figure. Each colored data point corresponds to the α exponent in a specific state: wakefulness (red), non-REM N2 (light blue), non-REM N3 (yellow), and REM (dark blue). The mean value is represented by a white-centered circle, and the standard deviation is indicated by the bounds of the shaded area, calculated for each sleep and wakefulness state. The data is arranged from left to right in ascending order based on the mean standard deviation of the DFA alpha across the four states, computed per region from all 60-s epochs and channels. Within the figure, data related to each region is organized into vertical blocks. Each block, arranged from left to right, includes the region's name along with the states Wakefulness, non-REM N2, non-REM N3, and REM. Below each block, there is a corresponding square in a distinct color, signifying the lobular region to which it belongs. Beneath each square, there is a number that identifies the region according to Table 1. The association of each color with its respective lobular region is depicted in the horizontally extended bar beneath the figure. The most significant variations are observed in the precentral and postcentral gyri, linked to motor and sensory functions. The cuneus region, associated with visual processing, also exhibit notable differences. The hippocampus displays the least variation, with REM sleep showing higher α values than non-REM states. Wakefulness α values are close to 1, while non-REM N2 and N3 states surpass 1.

Finally, although a full regional analysis is not the main objective here, to obtain a more complete picture of the DFA exponents α variability across different brain regions, the data from all analyzed channels were categorized by region and plotted in Figure 12. Each colored data point represents the exponent α in a specific brain region. The mean value (depicted by a white-centered circle) and the standard deviation (represented by the bounds of the shaded area) were calculated for each sleep and wakefulness state.

In Figure 12, the data are displayed from left to right in ascending order according to the mean standard deviation of the DFA alpha across the four states, calculated per region using all 60-s epochs and channels. Each region's data is presented in vertical blocks, including the region's name and states (Wakefulness, non-REM N2, N3, REM). Corresponding colored squares beneath each block indicate the lobular region. The color-region association is shown in the extended bar below the figure.

Upon inspecting Figure 12, notable variability in DFA exponent values is prominent in the precentral and postcentral gyri regions, associated with the motor and sensory cortices, respectively. Subsequently, is the cuneus region, which is situated in the posterior part of the occipital lobe, and stands out for its involvement in fundamental visual processing. At the opposite extreme of Figure 12 lies the hippocampus within the temporal lobe, which plays a pivotal role in behavior inhibition, memory, and spatial perception.

## 4 Discussion

### 4.1 Spectral dynamics across vigilance states

Consistent with established sleep physiology, we observed a marked increase in low-frequency (especially delta-band) power during NREM sleep compared to wakefulness. These slow oscillations (below 5 Hz) are hallmark features of deep NREM sleep and are most prominent in frontal–medial cortex, consistent with widespread synchrony observed in intracranial recordings (Brancaccio et al., 2020).

In parallel, sleep spindles (12–15 Hz), were particularly elevated in N2 (and attenuated in N3). Sleep spindles are characteristic of stage-N2 thalamocortical architecture (Staresina et al., 2023). Although we did not assess memory behaviorally, the delta–sigma distribution we observed matches canonical stage-specific spectral profiles.

Wakefulness, in contrast, was dominated by faster theta and alpha oscillations, with reduced delta power. This spectral profile reflects an externally oriented brain state, engaged in sensory processing and encoding of new information (Klimesch, 1999). REM sleep displayed a hybrid profile. Higher-order cortical areas showed fast activity, while primary sensory regions remained in a delta-rich state, consistent with the idea of sensory disconnection during REM (Hobson et al., 2000). This REM profile—fast activity in higher-order cortices alongside persistent limbic delta—is consistent with accounts that REM may protect internally generated reactivation from external interference while permitting the integration of recently acquired information (Hobson et al., 2000; Diekelmann and Born, 2010). In our data, visual regions such as the calcarine cortex showed reduced α-band power during REM stage, compared to wake, suggesting diminished visual input, while limbic areas behaved differently. The amygdala showed decreased delta power in REM (relative to wake), whereas the hippocampus maintained elevated delta power, hinting at continued internal replay or integration (von Ellenrieder et al., 2020).

These differences between wakefulness and REM sleep are particularly relevant—not only because they reflect distinct functional roles, but also because they may serve as robust, physiologically grounded spectro-anatomical markers for distinguishing the two states. Our findings—highlighting, for example, elevated alpha power in the primary visual cortex and increased delta activity in the amygdala during wake, as well as enhanced delta power in the hippocampus during REM—suggest that anatomically specific spectral features (e.g., occipital alpha versus limbic delta) could improve physiologically informed state classification in iEEG datasets.

Importantly, the observed elevation of medial-frontal delta in N3 relative to N2 aligns with findings by Bernardi et al. (2019), who documented source-localized slow-wave amplitude increases in frontal regions during deep sleep. This suggests regional slow-wave amplification as a marker of sleep depth rather than tissue-specific dysfunction. Similarly, the persistence of high delta power in the hippocampus during REM—contrasting with cortical desynchronization—parallels intracranial observations (Moroni et al., 2007; Ferrara et al., 2012), emphasizing a unique hippocampal signature of REM characterized by tonic delta synchronization.

Moreover, the decrease in amygdala delta during REM mirrors findings from emotion-related intracranial studies, where REM selectively attenuates amygdala reactivity overnight (e.g. Wassing et al., 2019). Such limbic divergence exemplifies functionally heterogeneous spectral modulation across subcortical structures.

Finally, the band-specific differences observed in beta and gamma between N2 and N3—particularly their reduction in N3 over fronto-parietal cortices—are consistent with Brancaccio et al. (2020). Taken together, these findings underscore that transitions from N2 to N3 are accompanied not only by slow-wave augmentation but also by broadband desynchronization of higher-frequency activity, possibly reflecting global network downscaling.

### 4.2 Neuronal avalanches

We analyzed neuronal avalanche size distributions across wakefulness, REM, and NREM sleep stages. A key finding was that the fitted power-law exponent τ varied systematically with vigilance state. Avalanche scaling analysis was performed using a fixed binning approach with a consistent detection threshold of −2.0 standard deviations. The size exponent (τ) ranged from 1.6–2.0 during N2/N3 sleep to 2.0–2.4 in wakefulness and REM states. This indicates a relative increase in the frequency of larger avalanches during wakefulness and REM compared to NREM sleep. Duration exponents (α) showed a clear progression, from 1.7–2.1 in NREM sleep to 2.3–2.9 in REM and wakefulness, reflecting longer avalanche lifetimes in more activated brain states. Spatial scaling (γ) exhibited state-dependent variations, with values of 1.1 in N3 sleep and 1.4 during wakefulness, consistent with increased spatial propagation and integration of avalanches during wakefulness. Furthermore, the increased Kolmogorov–Smirnov (KS) distance observed in power-law fitting during N3 indicates that in this state, avalanche size distributions deviate more significantly from an ideal power-law, suggesting reduced adherence to scale-free dynamics. These quantitative changes in scaling exponents reflect systematic modulations in the spatiotemporal organization of neuronal avalanches across sleep–wake states.

Our results align with prior intracranial and EEG studies showing that vigilance state modulates avalanche statistics (Priesemann et al., 2013).

Limitations in spatial sampling inherently bias the estimation of scaling exponents toward higher values, as previously reported studies (Priesemann et al., 2009). Despite this systematic upward shift, the relative differences in scaling exponents between vigilance states remain preserved and are more reliably distinguished due to the consistent sampling methodology applied across conditions. Consequently, spatial undersampling, while affecting absolute exponent values, does not compromise the comparative analysis of state-dependent avalanche dynamics.

That said, cortical networks during deep sleep are strongly shaped by slow oscillations and synchrony—features that can yield similar scaling without fine tuning. Empirical and modeling work on driven or adaptive networks shows that scale-invariant avalanche patterns can emerge without invoking a phase transition (Priesemann et al., 2014; Martinello et al., 2017; Lombardi et al., 2023). Accordingly, we keep our interpretation descriptive—as state-dependent variation in scaling—without committing to a specific underlying mechanism or operating regime.

Wakefulness and REM sleep showed higher τ values and fewer large avalanches, consistent with more fragmented scaling. This matches observations from MEG and EEG studies (Shriki et al., 2013) and may reflect a functional preference for adaptability and responsiveness over large-scale synchronization.

Our findings are also consistent with longitudinal rodent studies, which have reported a re-expansion of avalanche size distributions during sleep following wake-related shifts in scaling (Xu et al., 2024). These patterns are consistent with sleep-dependent network recalibration, but do not, on their own, demonstrate fine-tuned operation in any specific regime.

Neuronal avalanches have been proposed as signatures of scale-free activity in cortical networks, motivating extensive theoretical and empirical work (Chialvo, 2010; Beggs and Plenz, 2003). Our aim here is not to adjudicate between competing frameworks, but to characterize how avalanche size distributions vary systematically across sleep stages and how these shifts relate to state-dependent processing.

More recent models show that scale-invariant avalanche statistics can emerge in driven, neutral, or adaptive networks without fine tuning (Martinello et al., 2017; Matin et al., 2021; Lombardi et al., 2023). In light of this, we keep our interpretation descriptive—reporting empirical scaling relationships—without assigning them to a particular dynamical context.

### 4.3 Temporal correlations and information integration windows

DFA revealed state-dependent organization of neural dynamics. At intermediate timescales (0.04-2.5s), wakefulness and REM typically showed 0.6 < α < 1.0, consistent with stationary long-range temporal correlations (LRTC). In contrast, N2 and N3 exhibited α > 1, reflecting non-stationary, integrated persistence consistent with the predominance of slow oscillatory activity in NREM. These scale-dependent patterns align with previous EEG studies (Lee et al., 2002, 2004; Goshvarpour et al., 2013; Ma et al., 2018), and the iEEG dataset used in this study confirms their robustness across cortical and subcortical territories. We keep our analysis descriptive, without interpreting α within any particular theoretical framework.

These temporal regimes reflect a shift in the intrinsic correlation structure of neural dynamics across vigilance states. In wakefulness and REM, moderate long-range correlations suggest persistent but stationary activity patterns. The similarity between REM and wake in short-scale DFA profiles highlights shared dynamic features, despite their different sources of activation.

In NREM, α > 1 denotes non-stationary, integrated persistence, consistent with stronger slow-timescale autocorrelation during deep sleep (Zhang et al., 2021; Meisel et al., 2017).

Our region-specific DFA analysis further revealed heterogeneous modulations of temporal dynamics. While sensory and prefrontal cortices showed marked shifts in α between wakefulness and NREM, the hippocampal α remained comparatively stable across states (Tagliazucchi et al., 2013), a pattern compatible with uninterrupted internal reactivation in two-stage accounts, although we did not assess replay or behavioral memory outcomes.

These findings align with intracranial studies showing asynchronous NREM to REM transitions, starting in occipital regions before reaching frontal areas (Peter-Derex et al., 2023). They also reflect known increases in DFA variability during deep sleep (Goshvarpour et al., 2013). The current results provide a refined anatomical granularity, identifying specific regions with functionally meaningful dynamics.

Overall, our DFA findings demonstrate that sleep influences not only the spectral and spatial organization of neural activity, but also the intrinsic temporal architecture of cortical and subcortical dynamics. The scaling exponent α provides a compact summary of state-dependent changes in temporal structure, highlighting enhanced persistence during deep sleep and dynamic variability across brain regions.

### 4.4 Integration of multiscale dynamics and implications

By combining oscillatory analysis with avalanche statistics and scaling measures, this work highlights how multiple levels of neural organization collectively contribute to cognitive function across vigilance states. The convergent picture that emerges is that sleep is not a quiescent period, but rather an active neurophysiological process of reorganization—one that potentially lays the groundwork for memory consolidation and brain-wide plasticity.

Slow-wave sleep, in particular, appears to create the necessary conditions for stabilization and integration of new memory representations: strong delta oscillations could synchronize widespread networks, neuronal avalanches skew toward large-scale coactivations, and non-stationary, integrated persistence. These features are those expected to support the repeated reactivation of recently learned neuronal assemblies and the strengthening of synaptic connections according to Hebbian principles (Tononi and Cirelli, 2003; Diekelmann and Born, 2010).

REM sleep, on the other hand, appears to share many dynamical traits with wakefulness, yet it operates in an isolated environment shielded from external perturbations. This unique combination in REM may allow the brain to autonomously explore and refine neural representations—potentially integrating new memories with older networks, resolving interference, and modulating emotional tone—while maintaining a high level of network plasticity (Fagerholm et al., 2015).

Such a role is consonant with hypotheses that REM sleep supports memory consolidation in a complementary fashion to NREM (Diekelmann and Born, 2010).

### 4.5 Limitations

It is important to recognize the limitations of our interpretations. The links we draw between the observed neural signatures and memory functions are based on consistency with prior empirical and theoretical work (e.g., Tononi and Cirelli, 2003; Diekelmann and Born, 2010; Shew et al., 2009; Tagliazucchi et al., 2013). We thus refrain from making definitive claims that “memory consolidation occurred” in our participants; rather, we argue that the brain states we characterized are compatible with the neurophysiological requirements of consolidation as established in the literature. Notably, extensive research in animals has demonstrated causal connections between specific sleep phenomena and memory—for example, the work of Girardeau et al. (2009) showing that disrupting hippocampal ripples in NREM sleep impairs spatial memory, and evidence that enhancing slow oscillation–spindle coupling in humans can improve recall (Staresina et al., 2015).

These studies bolster the interpretation that the patterns we observe (e.g., abundant NREM slow oscillations with spindles, and an active hippocampus during REM) are indeed meaningful for memory processing. However, future studies should explicitly link our multiscale metrics to behavioral memory performance in order to confirm their functional significance.

Second, our data were obtained from individuals with drug-resistant epilepsy who had intracranial electrodes implanted for clinical monitoring. Although epileptic spikes and seizures were carefully excluded and focused on periods of normal sleep, the generalizability of the findings to healthy brains must be made with caution. Prior work suggests that many of the phenomena we report (such as spectral signatures of sleep stages and avalanche dynamics) have also been observed with noninvasive recordings in healthy populations (Tagliazucchi et al., 2013).

Nonetheless, the potential influence of pathological hyperexcitability or medication effects cannot be completely ruled out. We acknowledge this caveat and view our findings as a first step that should be replicated in further datasets, including those from healthy sleepers if intracranial recordings become available (e.g., during presurgical mapping in non-epileptic tumor cases or with emerging minimally invasive techniques).

Third, our conclusions on temporal correlations and avalanche distributions are based on empirical results validated by statistical significance tests. Linking these patterns to specific theoretical models is beyond the scope of this work.

In all cases, we emphasize a cautious approach: the multiscale descriptors we report are robust and novel characterizations of sleep–wake states, but their exact roles in memory processing remain to be fully determined.

### 4.6 Conclusions and future directions

This work demonstrates the power of a multiscale analytic approach to sleep neurophysiology, combining spectral, temporal, and network-level assessments to reveal how the sleeping brain balances the demands of information storage and responsiveness.

Beyond physiological interpretation, our findings also highlight opportunities for feature-engineered sleep staging. Region- and band-specific spectral signatures (e.g., posterior alpha in primary visual cortex; delta modulation in limbic and frontal areas) provide physiologically grounded inputs that can aid automated discrimination—particularly for the challenging pairs Wake vs. REM and N2 vs. N3. Complementing these with local temporal descriptors from DFA—specifically the intermediate-scale exponent α (0.04–2.5 s) computed per region, which tends to be ~0.6-1.0 in Wake/REM and >1 in N2/N3—yields a compact, interpretable feature set. Integrating (i) Gaussian-fitted band amplitudes and (ii) region-wise DFA α (optionally alongside spatial co-activation metrics) could improve classifier performance while keeping models grounded in neurophysiology and tractable on 60-s epochs.

On the other hand, future studies should aim to link these multiscale neural signatures with behavioral measures of memory performance, and extend these analyses to larger and more diverse populations, including healthy individuals. Furthermore, collaborative efforts between clinical researchers, computational neuroscientists, and sleep specialists will be essential for uncovering the full cognitive relevance of these dynamic sleep states. The integration of iEEG with interventional techniques such as stimulation or pharmacological modulation may help establish causal links between the observed neural patterns and memory outcomes.

Looking ahead, future efforts could focus on patient-specific modeling and predictive analysis, particularly in clinical contexts where disrupted sleep impacts cognitive function. Establishing direct links between iEEG dynamics and behavioral or memory task performance will be key to clarifying their functional relevance. Additionally, incorporating spatially-aware machine learning methods—such as graph neural networks—may enhance our ability to decode inter-regional interactions. Extending these approaches to longitudinal and developmental datasets could provide new insights into how sleep-related neural representations evolve across the lifespan or in pathological conditions.

## Data Availability

Publicly available datasets were analyzed in this study. This data can be found here: https://ieegatlas.loris.ca/.
